# West Nile Virus and Other Nationally Notifiable Arboviral Diseases — United States, 2022

**DOI:** 10.15585/mmwr.mm7321a2

**Published:** 2024-05-30

**Authors:** Rebekah A. Sutter, Shelby Lyons, Carolyn V. Gould, J. Erin Staples, Nicole P. Lindsey

**Affiliations:** ^1^Division of Vector-Borne Diseases, National Center for Emerging and Zoonotic Infectious Diseases, CDC; ^2^Epidemic Intelligence Service, CDC.

**Summary**

**What is already known about this topic?**

Humans become infected by arboviruses primarily through the bite of an infected mosquito or tick. West Nile virus is the leading cause of arboviral disease in the continental United States.

**What is added by this report?**

Despite fewer total arboviral disease cases in 2022 compared with 2021, historically high numbers of St. Louis encephalitis and Powassan virus disease cases were reported.

**What are the implications for public health practice?**

The variable occurrence of arboviral diseases highlights the importance of surveillance efforts in targeting prevention messaging and control. Health care providers should consider arboviral testing for patients with clinically compatible illnesses. Prevention depends on reducing vector populations, implementing personal protective measures to decrease exposure, and screening blood and tissue donors.

## Abstract

Arthropodborne viruses (arboviruses) primarily infect humans through the bite of an infected mosquito or tick. Infections are commonly asymptomatic; however, the clinical signs and symptoms can range from a mild febrile illness to severe neuroinvasive disease. This report summarizes data for six nationally notifiable arboviral diseases for 2022 reported to ArboNET, the national surveillance system for arboviral diseases, including eastern equine encephalitis, Jamestown Canyon, La Crosse, Powassan, St. Louis encephalitis, and West Nile viruses. In 2022, these viruses caused 1,247 human disease cases, 968 (78%) hospitalizations, and 103 (8%) deaths. Reported case counts decreased from 2021 for all viruses except Powassan and St. Louis encephalitis viruses. Despite a substantial decrease in reported cases from 2021, West Nile virus remained the leading cause of arboviral disease in the continental United States. Variations in annual arboviral disease incidence and distribution highlight the importance of high-quality surveillance. Health care providers should suspect arboviral infection in patients with a clinically compatible illness, consider testing, and report positive findings to their state or local health department. In areas with arboviral activity, community and household efforts to reduce vector populations (e.g., applying insecticides and reducing breeding sites) and personal protective measures to decrease mosquito and tick exposures (e.g., wearing repellents and protective clothing) can reduce arboviral disease morbidity and mortality.

## Introduction

Arthropodborne viruses (arboviruses) are transmitted to humans primarily through the bite of an infected mosquito or tick. Rarely, transmission occurs through blood transfusion and organ transplantation. West Nile virus (WNV) is the leading cause of arboviral disease in the continental United States (*1*). Other domestic arboviruses cause sporadic cases and occasional outbreaks. Most arboviral infections are asymptomatic, with clinical signs and symptoms ranging from a mild febrile illness to severe neuroinvasive disease (*2*). This report summarizes nationally notifiable arboviral diseases reported to CDC for 2022.

## Methods

Data for six nationally notifiable, domestic arboviruses (eastern equine encephalitis, Jamestown Canyon, La Crosse, Powassan, St. Louis encephalitis, and West Nile viruses) were analyzed and are included in this report. Chikungunya, dengue, yellow fever, and Zika virus disease cases are excluded because these infections are primarily travel-associated when they occur in U.S. states (*3*,*4*). Surveillance data are obtained from ArboNET, the national surveillance system for arboviral diseases. Disease cases are reported by state health departments to ArboNET using a standard case definition that includes clinical and laboratory criteria.* Cases reported as probable^†^ and confirmed^§^ are included in this report and are reported on the basis of state and county of residence. Cases are described by demographic characteristics including age and sex, quarter year of illness onset (January–March, April–June, July–September, and October–December), clinical syndrome (neuroinvasive [acute flaccid paralysis, encephalitis, meningitis, or other neurologic signs and symptoms] versus nonneuroinvasive [all other cases]), and outcome (hospitalization and death). Incidence was calculated using 2022 midpoint population estimates from the U.S. Census Bureau.^¶^ All statistical analyses were conducted using SAS software (version 9.4; SAS Institute). This activity was reviewed by CDC, deemed not research, and was conducted consistent with applicable federal law and CDC policy.**

## Results

A total of 1,247 domestic arboviral disease cases with illness onset in 2022 were reported to CDC (Table 1). Overall, 1,132 (91%) cases were caused by WNV, followed by Powassan (47; 4%), St. Louis encephalitis (33; 3%), La Crosse (22; 2%), Jamestown Canyon (12; 1%), and eastern equine encephalitis (one; <1%) viruses. Cases were reported from 414 (13%) of the 3,143 U.S. counties in 45 states and the District of Columbia (DC).

**TABLE 1:** Number and percentage of reported cases of nationally notifiable nonneuroinvasive and neuroinvasive arboviral diseases, by virus type and selected patient characteristics (N = 1,247)* — United States, 2022

Characteristic	Virus type, no. (%) of cases				
	West Nile n = 1,132	Powassan n = 47	St. Louis encephalitis n = 33	La Crosse n = 22	Jamestown Canyon n = 12
**Age group, yrs**					
<18	25 (2)	7 (15)	0 (—)	21 (95)	0 (—)
18–59	429 (38)	14 (30)	14 (42)	0 (—)	6 (50)
≥60	678 (60)	26 (55)	19 (58)	1 (5)	6 (50)
Median age (IQR)	63 (50–73)	64 (43–72)	65 (50–74)	9 (5–11)	60 (40–74)
**Sex**					
Female	439 (39)	21 (45)	13 (39)	10 (45)	3 (25)
Male	693 (61)	26 (55)	20 (61)	12 (55)	9 (75)
**Period of illness onset^†^**					
Jan–Mar	9 (1)	4 (9)	0 (—)	0 (—)	1 (8)
Apr–Jun	36 (3)	21 (45)	5 (15)	0 (—)	5 (42)
Jul–Sep	966 (85)	11 (23)	15 (45)	20 (91)	2 (17)
Oct–Dec	120 (11)	11 (23)	13 (39)	2 (9)	4 (33)
**Clinical syndrome**					
Nonneuroinvasive	305 (27)	4 (9)	6 (18)	3 (14)	1 (8)
Neuroinvasive	827 (73)	43 (91)	27 (82)	19 (86)	11 (92)
Encephalitis^§^	501 (61)	29 (67)	14 (52)	16 (84)	6 (55)
Meningitis^§^	210 (25)	4 (9)	10 (37)	3 (16)	3 (27)
AFP^§,¶,^**	41 (5)	4 (9)	1 (4)	0 (—)	0 (—)
Unspecified^§^	75 (9)	6 (14)	2 (7)	0 (—)	2 (18)
**Outcome**					
Hospitalization	862 (76)	45 (96)	29 (88)	21 (95)	10 (83)
Death	93 (8)	7 (15)	3 (9)	0 (—)	0 (—)

**Abbreviation**: AFP = acute flaccid paralysis.

* One eastern equine encephalitis virus disease case was also reported.

^†^ Date of illness onset is unknown for one case of West Nile virus disease.

^§^ Percentages of cases of encephalitis, meningitis, AFP, and unspecified neurologic signs or symptoms are percentages of neuroinvasive cases.

^¶^ Among the 41 West Nile virus disease cases with AFP, 10 (24%) also had encephalitis or meningitis.

** Among the four Powassan virus disease cases with AFP, three also had encephalitis or meningitis.

### West Nile Virus Disease

The 1,132 WNV disease cases were reported from 358 counties in 42 states and DC; 966 (85%) patients had illness onset during July–September. Median patient age was 63 years, and 61% were male. A total of 862 (76%) patients were hospitalized, and 93 (8%) died. Three patients with nonfatal neuroinvasive disease were infected through solid organ transplants from a common donor.

Among all patients with WNV disease, 827 (73%) had neuroinvasive disease, 772 (93%) of whom were hospitalized, including 91 (11%) who died. The national incidence of neuroinvasive WNV disease was 0.25 per 100,000 population (Table 2). The highest WNV neuroinvasive disease incidences occurred in South Dakota (3.96 per 100,000), Colorado (2.24), and Nebraska (1.88). The largest numbers of neuroinvasive disease cases were reported from California (162), Colorado (131), and New York (75), accounting for 44% of neuroinvasive disease cases nationally. WNV neuroinvasive disease incidence increased with age from 0.01 per 100,000 among persons aged <10 years to 0.78 per 100,000 among those aged ≥70 years. Incidence of WNV neuroinvasive disease was 68% higher among males (0.32 per 100,000) than among females (0.19).

**TABLE 2:** Number and incidence* of reported cases of nationally notifiable arboviral neuroinvasive disease, by virus type and U.S. Census Bureau division and jurisdiction — United States, 2022

U.S. Census Bureau division/jurisdiction	Neuroinvasive disease cases, by virus type, no. (incidence)*				
	West Nile	Powassan	St. Louis encephalitis	La Crosse	Jamestown Canyon
**United States**	**827 (0.25)**	**43 (0.01)**	**27 (<0.01)**	**19 (<0.01)**	**11 (<0.01)**
**New England**	15 (0.10)	15 (0.10)	—^†^	—	3 (0.02)
Connecticut	7 (0.19)	6 (0.17)	—	—	—
Maine	—	4 (0.29)	—	—	—
Massachusetts	7 (0.10)	4 (0.06)	—	—	1 (0.01)
New Hampshire	—	—	—	—	—
Rhode Island	1 (0.09)	—	—	—	2 (0.18)
Vermont	—	1 (0.15)	—	—	—
**Middle Atlantic**	114 (0.27)	13 (0.03)	—	—	—
New Jersey	13 (0.14)	2 (0.02)	—	—	—
New York	75 (0.38)	7 (0.04)	—	—	—
Pennsylvania	26 (0.20)	4 (0.03)	—	—	—
**East North Central**	57 (0.12)	8 (0.02)	—	12 (0.03)	7 (0.01)
Illinois	27 (0.21)	—	—	—	—
Indiana	6 (0.09)	—	—	—	—
Michigan	13 (0.13)	—	—	—	3 (0.03)
Ohio	5 (0.04)	—	—	12 (0.10)	—
Wisconsin	6 (0.10)	8 (0.14)	—	—	4 (0.07)
**West North Central**	123 (0.57)	7 (0.03)	—	3 (0.10)	1 (<0.01)
Iowa	8 (0.25)	—	—	—	—
Kansas	6 (0.20)	—	—	—	—
Minnesota	17 (0.30)	7 (0.12)	—	3 (0.05)	1 (0.02)
Missouri	11 (0.18)	—	—	—	—
Nebraska	37 (1.88)	—	—	—	—
North Dakota	8 (1.03)	—	—	—	—
South Dakota	36 (3.96)	—	—	—	—
**South Atlantic**	59 (0.09)	—	—	3 (<0.01)	—
Delaware	1 (0.10)	—	—	—	—
District of Columbia	1 (0.15)	—	—	—	—
Florida	7 (0.03)	—	—	—	—
Georgia	16 (0.15)	—	—	—	—
Maryland	6 (0.10)	—	—	—	—
North Carolina	12 (0.11)	—	—	2 (0.02)	—
South Carolina	10 (0.19)	—	—	—	—
Virginia	6 (0.07)	—	—	—	—
West Virginia	—	—	—	1 (0.06)	—
**East South Central**	17 (0.09)	—	—	1 (<0.01)	—
Alabama	6 (0.12)	—	—	—	—
Kentucky	3 (0.07)	—	—	—	—
Mississippi	5 (0.17)	—	—	—	—
Tennessee	3 (0.04)	—	—	1 (0.01)	—
**West South Central**	87 (0.21)	—	1 (<0.01)	—	—
Arkansas	3 (0.10)	—	—	—	—
Louisiana	41 (0.89)	—	—	—	—
Oklahoma	4 (0.10)	—	—	—	—
Texas	39 (0.13)	—	1 (<0.01)	—	—
**Mountain**	187 (0.73)	—	12 (0.05)	—	—
Arizona	40 (0.54)	—	12 (0.16)	—	—
Colorado	131 (2.24)	—	—	—	—
Idaho	1 (0.05)	—	—	—	—
Montana	—	—	—	—	—
Nevada	—	—	—	—	—
New Mexico	8 (0.38)	—	—	—	—
Utah	5 (0.15)	—	—	—	—
Wyoming	2 (0.34)	—	—	—	—
**Pacific**	168 (0.32)	—	14 (0.03)	—	—
Alaska	—	—	—	—	—
California	162 (0.42)	—	14 (0.04)	—	—
Hawaii	—	—	—	—	—
Oregon	3 (0.07)	—	—	—	—
Washington	3 (0.04)	—	—	—	—

* Cases per 100,000 population, based on July 1, 2022, U.S. Census Bureau population estimates.

^†^ Dashes indicate no reported cases.

### Powassan Virus Disease

Forty-seven cases of Powassan virus disease were reported from 39 counties in nine states. In 2022, Powassan virus disease was reported from Vermont for the first time. Illness onset occurred most frequently during April–June (45%) (Table 1). Median patient age was 64 years, and 55% of patients were male. Forty-three (91%) patients experienced neuroinvasive disease, 45 (96%) patients were hospitalized, and seven (15%) died. States with the highest incidence of neuroinvasive disease included Maine (0.29 per 100,000), Connecticut (0.17), and Vermont (0.15) (Table 2). All patients who died were aged >60 years (median age = 67 years; range = 61–91 years).

### St. Louis Encephalitis Virus Disease

Thirty-three cases of St. Louis encephalitis virus disease were reported from 12 counties in three states. Illness onset occurred most frequently during July–September (45%), although 39% of cases occurred during October–December (Table 1). All late-season cases were reported in the southwestern United States (Arizona, California, and Texas). Median patient age was 65 years, and 61% of patients were male. Twenty-seven (82%) patients had neuroinvasive disease, 29 (88%) were hospitalized, and three (9%) died. The highest incidences of neuroinvasive disease were reported from Arizona (0.16 per 100,000) and California (0.04) (Table 2). All patients who died were aged >65 years (median age = 83 years; range = 68–85 years).

### La Crosse Virus Disease

Twenty-two cases of La Crosse virus disease were reported from 19 counties in five states. Twenty (91%) patients experienced illness onset during July–September (Table 1); the median patient age was 9 years, and 55% of patients were male. Nineteen (86%) patients had neuroinvasive disease. Twenty-one (95%) patients were hospitalized; none died. Ohio reported the highest number of neuroinvasive disease cases (12; 63%) (Table 2), and the highest incidences of neuroinvasive disease occurred in Ohio (0.10 per 100,000), West Virginia (0.06), and Minnesota (0.05).

### Jamestown Canyon Virus Disease

Among 12 cases of Jamestown Canyon virus disease reported from 12 counties in five states, illness onset occurred most frequently during April–June (five cases) (Table 1). The median patient age was 60 years, and nine of the 12 patients were male. All but one patient had neuroinvasive disease and 10 patients were hospitalized; no deaths were reported. Wisconsin reported the highest number of neuroinvasive disease cases (four) and the highest incidence (0.18 per 100,000) of neuroinvasive disease occurred in Rhode Island (Table 2).

### Eastern Equine Encephalitis Virus Disease

One case of eastern equine encephalitis virus disease was reported. The patient was a woman aged >60 years with illness onset in August. The patient experienced neuroinvasive disease and was hospitalized.

## Discussion

Overall, the number of arboviral disease cases reported in 2022 (1,247) decreased 59% compared with the 3,035 cases reported in 2021. This decrease was largely driven by a 61% decrease in reported WNV disease cases in 2022 (1,132) compared with the 2,911 cases reported in 2021, when a large WNV disease outbreak occurred in Arizona (*1*). WNV disease remained the most commonly reported domestic arboviral disease. La Crosse virus remained the most common cause of neuroinvasive arboviral disease in children.

In contrast to other arboviruses, historically high numbers of St. Louis encephalitis virus and Powassan virus disease cases were reported in 2022. The 33 St. Louis encephalitis virus disease cases represent the highest number of cases since 2003, when 49 cases were reported (*5*). The 47 Powassan virus disease cases represent the highest number ever reported in a single year; the previous high was 43 cases reported in 2019 (*6*).

Arboviral diseases remain an important cause of morbidity in the United States. Although most human infections occur through the bite of infected mosquitoes or ticks, organ transplant transmission continues to occur. Currently, no national policy exists requiring arboviral screening of deceased donors (*7*). The complex interaction among humans, animals, and environment that contributes to vectorborne transmission poses challenges to predicting and controlling disease. Timely and high-quality surveillance (e.g., accurate and complete case identification, investigation, and reporting) is important to detecting arboviral disease risk and implementing interventions to lower disease incidence such as distributing prevention messaging and performing vector control activities.

### Limitations

The findings in this report are subject to at least two limitations. First, ArboNET is a passive surveillance system and, as such, likely underestimates disease prevalence. Identifying cases via ArboNET relies on patients seeking health care, providers ordering testing and establishing a diagnosis, and laboratories and clinicians reporting cases to local and state health departments. Nonneuroinvasive disease reporting is more susceptible to underreporting because patients might not seek care or undergo arboviral testing. Previous studies have estimated that 30–70 nonneuroinvasive disease cases occur for every reported case of West Nile neuroinvasive disease (*8*). Based on the 827 neuroinvasive disease cases reported in 2022, an estimated 24,810–57,890 nonneuroinvasive disease cases occurred; however, only 305 (0.5%–1.2% of the estimated total) were reported. Second, because ArboNET does not require information about clinical signs and symptoms or laboratory findings, cases might be misclassified, potentially affecting case classification.

### Implications for Public Health Practice

Understanding the epidemiology, seasonality, and geographic distribution of arboviruses is important for clinical recognition. Health care providers should consider arboviral disease testing in patients with a clinically compatible illness (e.g., febrile illness, meningitis, or encephalitis) during transmission seasons when ticks and mosquitos are active or after receipt of transplanted organs or blood transfusion. Positive test results should be reported to a state or local health department. Infections temporally associated with blood transfusion or organ transplantation should also be reported promptly to allow potentially infected products to be identified and removed from circulation. No treatments or vaccines are currently available for domestic arboviral infections. Therefore, prevention and control efforts rely on personal protective measures to decrease exposure to mosquitos^††^ and ticks^§§^ (e.g., wearing repellents and protective clothing), community and household effort to decrease vector populations,^¶¶^ (e.g., applying insecticides and reducing breeding sites), and blood donor screening to minimize transfusion transmission.***

## Footnotes

*
https://ndc.services.cdc.gov/case-definitions/arboviral-diseases-neuroinvasive-and-non-neuroinvasive-2015/

† A probable case meets clinical criteria for arboviral infection and virus-specific immunoglobulin M (IgM) antibodies in cerebrospinal fluid (CSF) or serum but without other testing.

§ A confirmed case meets clinical criteria for arboviral disease and at least one of the following laboratory criteria: 1) isolation of virus from, or demonstration of specific viral antigen or nucleic acid in, tissue, blood, CSF, or other body fluid; 2) fourfold or greater change in virus-specific quantitative antibody titers in paired sera; 3) virus-specific IgM antibodies in serum with confirmatory virus-specific neutralizing antibodies in the same or a later specimen; or 4) virus-specific IgM antibodies in CSF and a negative result for other IgM antibodies in CSF for arboviruses endemic in the region where exposure occurred.

¶
https://www.census.gov/data/tables/time-series/demo/popest/2020s-state-total.html

** 45 C.F.R. part 46, 21 C.F.R. part 56; 42 U.S.C. Sect. 241(d); 5 U.S.C. Sect. 552a; 44 U.S.C. Sect. 3501 et seq.

††
https://www.cdc.gov/mosquitoes/prevention/index.html

§§
https://www.cdc.gov/ticks/prevention/index.html

¶¶
https://www.cdc.gov/mosquitoes/mosquito-control/index.html

***
https://www.cdc.gov/blood-safety/about/index.html
